# Anthropometry and bone mineral density in treated and untreated hyperphenylalaninemia

**DOI:** 10.1530/EC-20-0214

**Published:** 2020-06-08

**Authors:** Mojca Zerjav Tansek, Ana Bertoncel, Brina Sebez, Janez Zibert, Urh Groselj, Tadej Battelino, Magdalena Avbelj Stefanija

**Affiliations:** 1 Department of Pediatric Endocrinology, Diabetes and Metabolic Diseases, University Children’s Hospital, UMC Ljubljana, Ljubljana, Slovenia; 2 University of Ljubljana, Faculty of Medicine, Ljubljana, Slovenia; 3 Centre for Health Informatics and Statistics, Faculty of Health Sciences, University of Ljubljana, Ljubljana, Slovenia

**Keywords:** phenylketonuria, phenylalanine, body mass index, growth, final height

## Abstract

Despite recent improvements in the composition of the diet, lower mineral bone density and overweight tendencies are incoherently described in patients with phenylketonuria (PKU). The impact of dietary factors and plasma phenylalanine levels on growth, BMI, body composition, and bone mineral density was investigated in our cohort of patients with hyperphenylalaninemia (HPA) with or without dietary treatment. The anthropometric, metabolic, BMI and other nutritional indicators and bone mineral density were compared between the group of 96 treated patients with PKU (58 classic PKU (cPKU) and 38 patients with moderate-mild PKU defined as non-classic PKU (non-cPKU)) and the untreated group of 62 patients with benign HPA. Having compared the treated and untreated groups, there were normal outcomes and no statistically significant differences in BMI, body composition, and bone mineral density. Lower body height standard deviation scores were observed in the treated as compared to the untreated group (*P* < 0.001), but the difference was not significant when analyzing patients older than 18 years; however, cPKU adults were shorter compared to non-cPKU treated adults (*P* = 0.012). Interestingly, the whole-body fat was statistically higher in non-cPKU as compared to cPKU patients. In conclusion, the dietary treatment ensured adequate nutrition without significant consequences in BMI, body composition, and bone mineral density. A low protein diet may have delayed the growth in childhood, but the treated patients gained a normal final height. Mild untreated hyperphenylalaninemia characteristic for benign HPA had no negative physiological effect on bone mineral density.

## Introduction

Phenylketonuria (PKU) (OMIM 261600) is an autosomal recessive genetic disease caused by insufficient activity of the enzyme phenylalanine hydroxylase, which catalyzes the transformation of phenylalanine (Phe) to tyrosine (Tyr). The consequence of such disorder is an elevated serum concentration of Phe, that is, hyperphenylalaninemia (HPA), which, depending on the level of diminished enzyme activity, leads to irreversible damages of the central nervous system (CNS). For many years the only available treatment to prevent severe neurologic consequences of an enzymatic deficit was an early onset highly protein restrictive diet and the medical formula with Phe-free amino acids mixture, supplemented with minerals, trace elements and vitamins (1). Dietary treatment remains the mainstay of therapy for PKU, but this partially changed with the approval of a cofactor therapy. Sapropterin is a synthetic form of the tetrahydrobiopterin, which functions as a chaperone and stabilizes or activates unstable mutant enzyme phenylalanine hydroxylase, but this therapy is preferentially successful in milder forms of PKU (2). Newly available enzyme substitution therapy with modified phenylalanine ammonia lyase – pegvaliase – is approved for the treatment of adult patients with uncontrolled blood Phe concentrations, but the safety profile is not without concerns (3).

The natural protein intake of high biologic value is very low in PKU diet and the majority of the recommended protein intake is from the medical foods, which are rich in amino acids. With amino acid intake, the protein equivalent is increased to 140% or more of the recommended daily requirement established for healthy individuals, because of poor retention (4, 5). The patients, who are completely adherent to their daily medical food consumption recommendation, should not be at risk for nutrient deficiencies nowadays (5). The historical cohorts of the patients with PKU were not optimally treated in the past. They had insufficient blood levels of long-chain polyunsaturated fatty acids and selenium and the inclusion of these nutrients in the medical foods or as additional supplementation are warranted (6, 7).

Lower bone mineral density (BMD) and overweight tendency in PKU patients are described in some studies (4, 8), but not in the others (9, 10, 11), and it is unclear whether these observations could be a consequence of the diet composition or the metabolic disorder by itself. Recently, compliance with medical food prescription was shown to be a major positive predictor of BMD (12). Therapy with sapropterin enables increased and more relaxed natural protein intake, which is associated with a better BMD outcome (13).

Different forms of HPA have been described and different clinical phenotypes regarding the severity of the disease were previously classified as treated classic PKU (cPKU with Phe tolerance <20 mg/kg/day or pre-treatment blood Phe levels over 1200 µmol/L), moderate or variant PKU and mild HPA as non-classic PKU (non-cPKU) and untreated benign HPA (bHPA) (14). These criteria are no longer helpful for various reasons, including the early time of the neonatal screening and difficulties to determine exact Phe tolerance. The simplified classification in the most recent guidelines divides patients into groups requiring or not requiring treatment (4, 5). In our study, the treated group was further divided into cPKU and non-cPKU groups to evaluate the importance of low natural protein intake in a more detailed manner.

In Slovenia, neonatal screening for PKU was initiated in 1979 and facilitated the introduction of dietary treatment in the neonatal period for all patients (15, 16). The nationwide cohort of patients with PKU and non-cPKU are treated at a single national reference center for inborn errors of metabolism at University Children’s Hospital in Ljubljana.

Due to incoherent findings in different studies, we aimed to assess the impact of dietary treatment and metabolic phenotype on growth, body composition, and BMD in our cohort of patients with HPA.

## Materials and methods

### Subjects

The study was conducted between August 2015 and January 2017. Altogether 158 patients (60 adults and 98 children) with HPA of various severity were included in the retrospective study. The study was approved by the National Medical Ethics committee (No. 157/01/13, date 05.02.2013). Informed consent was obtained from parents of participants or adult participants before the study. All participants were diagnosed in the neonatal period and followed at a single center. According to the severity of HPA, the participants were divided into the treated group with 96 patients (cPKU and non-cPKU patients, 50 females, 48 adults, mean age 19 ± 10.7 years) and the untreated bHPA group with 62 patients (22 females, 8 adults, mean age 11.7 ± 6.8 years), who were diagnosed with neonatal screening with peak Phe below 360 µmol/L and did not need diet (Table 1). The treated group with cPKU consisted of 58 patients (30 females, 32 adults, mean age 24.3 ± 12 years) and non-cPKU of 38 patients (20 female, 16 adults, mean age 19.1 ± 9.7 years) (Table 2).

**Table 1:** Comparison of the characteristics of treated and untreated patients.

	Treated HPA^a^ (*n* = 96)	Untreated HPA^b^ (*n* = 62)	*P*-value
Age at DXA measurement (years)	19.0 ± 10.79	11.7 ± 6.8	<0.001
Age (years) (% age >18 years)	22.2 ± 11.4 (50%)	14.4 ± 6.8 (13%)	<0.001
Gender (M/F)	46/50	40/22	0.041
Anthropometry			
BMI SDS	0.8 ± 1.0	0.9 ± 1.1	0.86
Proportion of abdominal fat (%)	22.7 ± 7.8	21.1 ± 7.2	0.204
Proportion of whole-body fat (%)	25.8 ± 6.8	25.4 ± 6.7	0.758
Body height SDS	−0.06 ± 1.12	0.64 ± 1.17	<0.001
Body height SDS (age > 18 years)	−0.38 ± 1.15	−0.02 ± 1.42	0.425
Metabolic indicators			
Mean serum Phe (µmol/L)	465 ± 266	191 ± 80	<0.001
Maximum serum Phe (µmol/L)	1168 ± 481	282 ± 121	<0.001
Mean serum Tyr (µmol/L)	60 ± 13	66 ± 17	0.064
Mean Phe/Tyr ratio	10.3 ± 8.3	1.8 ± 1.8	<0.001
Nutritional indicators			
Phe intake tolerance (mg)	546 ± 236		
Serum B12 (pmol/L)	479 ± 198	428 ± 194	0.087
BMD			
Mean BMD (L1–L4) T- and Z-scores^c^	−0.54 ± 0.92	−0.40 ± 1.01	0.371
Mean BMD (total body) T- and Z-scores^c^	−0.66 ± 0.86	−0.24 ± 0.86	0.371

^a^Patients with classic and non-classical phenylketonuria; ^b^Patients with benign hyperphenylalaninemia; ^c^T-scores for adults and Z-scores for pediatric population were adjusted for the patient’s sex and age and compared with the control groups according to International Society for Clinical Densitometry guidelines (19).

B12, vitamin B12; BMD, bone mineral density; DXA, densitometry; F, female; HPA, hyperphenylalaninemia; L1–L4, first to forth lumbar vertebra; M, male; *n*, number; Phe, phenylalanine; SDS, standard deviation score; Tyr, tyrosin.

**Table 2:** Comparison of the characteristics of the treated patients with classic phenylketonuria (cPKU) and the rest of the patients with dietary treatment.

	Patients with classic PKU (*n* = 58)	Patient with non-classic PKU^a^ (*n* = 38)	*P*-value
Demographical data			
Age at DXA measurement (years)	20.9 ± 11.27	16.1 ± 9.4	0.05
Age (years) (% age >18 years)	24.3 ± 12.0 (55%)	19.1 ± 9.7 (42%)	0.05
Gender (M/F)	28/30	18/20	0.93
Anthropometry			
BMI SDS	0.7 ± 1.1	1.0 ± 1.0	0.11
Proportion of abdominal fat (%)	21.5 ± 7.9	23.9 ± 7.7	0.21
Proportion of whole-body fat (%)	24.3 ± 6.4	27.6 ± 6.9	0.04
Body height SDS	−0.40 ± 1.05	0.43 ± 1.04	<0.001
Body height SDS (age >18 years)	−0.63 ± 1.09	0.13 ± 0.99	0.012
Metabolic indicators			
Mean serum Phe (µmol/L)	560 ± 302	320 ± 72	<0.001
Maximum serum Phe (µmol/L)	1429 ± 416	769 ± 243	<0.001
Mean serum Tyr (µmol/L)	60 ± 13	60 ± 14	0.874
Mean Phe/Tyr ratio	13 ± 9	6 ± 3	<0.001
Nutritional indicators			
Phe intake tolerance (mg)	446 ± 128	696 ± 280	<0.001
Serum B12 (pmol/L)	530 ± 174	400 ± 209	<0.001
Serum zinc (µmol/L)	10.7 ± 1.8	10.1 ± 1.6	0.137
Serum selenium (µmol/L)	64.4 ± 15.8	68.1 ± 13.9	0.258
BMD			
Mean BMD (L1–L4) T- and Z-scores^b^	−0.60 ± 0.82	−0.45 ± 1.05	0.425
Mean BMD (total body) T- and Z-scores^b^	−0.73 ± 0.91	−0.57 ± 0.80	0.434

^a^Patients with variant PKU and mild hyperphenylalaninemia (non-classic PKU); ^b^T-scores for adults and Z-scores for pediatric population are adjusted for the patient’s sex and age and compared with the control groups according to International Society for Clinical Densitometry guidelines (19).

B12, vitamin B12; BMD, bone mineral density; DXA, densitometry; F, female; L1–L4, first to forth lumbar vertebra; M, male; *n*, number; Phe, phenylalanine; PKU, phenylketonuria; SDS, standard deviation score; Tyr, tyrosin.

### Anthropometry, biochemical methods and bone mineral density measurement

The anthropometric (body height, body mass index (BMI), proportion of whole-body and abdominal fat), metabolic (mean and maximum serum Phe, mean serum Tyr, mean Phe/Tyr ratio) and nutritional (Phe intake tolerance as an indicator of natural protein intake, serum B12 vitamin, zinc, and selenium), as well as BMD of lumbar spine and total body using dual-energy X-ray absorptiometry (DXA), expressed in g/cm^2^, were measured. All metabolic parameters available in the patient’s files were included in the study and mean values were calculated for each patient. Anthropometry, serum B12 vitamin, zinc, and selenium determined at the time of DXA measurement were included in the study. Results for BMI and body height were expressed as mean BMI standard deviation score (SDS) and body height SDS, which were calculated using the LMS method and the British 1990 reference growth data (17, 18) Anthropometric measurements were performed by a trained nurse using professional certified digital scales type Bolero (Arjo, Malmö, Sweden) and Digital Stadiometer 700–1600, QuickMedical® (Warwick, RI, USA).

Phe and Tyr levels were measured with a quantitative amino acid analyzer (HPLC Series 1200, USA with Pinnacle PCX, Pickering Laboratories, USA). The concentrations of selenium and zinc in plasma were determined by electrothermal atomic absorption spectrometry (ETAAS) and flame atomic absorption spectrometry (FAAS), respectively. A chemiluminescence method was used for vitamin B12 level determination.

The total body and lumbar spine (L1–L4) BMD was measured by dual-energy X-ray absorptiometry (Hologic, Discovery A DXA System (2272)). BMD was determined in grams per square centimetre (g/cm^2^); however, for statistical analysis, the T- (adults) and Z-scores (children and adolescents) were used, which are values adjusted for the patient’s sex and age and compared with the control groups according to International Society for Clinical Densitometry guidelines (19).

### Statistical analysis

The data between groups (Tables 1 and 2) were compared with the independent samples *t*-test in case of normally distributed data and with the Mann–Whitney *U*-test in the non-normally distributed case, while the differences between groups for categorized data were tested with the Chi-square test. The normality of data was checked with the Shapiro–Wilk test. The data in Tables 1 and 2 are presented in terms of mean ± SDS. A significant difference was considered when the *P*-value was less than 0.05.

The metabolic predictors of clinical characteristics of the treated patients in Table 3 were estimated by performing multiple step-wise linear regression with standardized regression coefficients (β). Included dependent variables were BMI SDS, the proportion of abdominal fat, the proportion of whole-body fat, the proportion of abdominal fat/proportion of whole-body fat ratio, serum vitamin B12, selenium and zinc, Z-scores and T-scores for mean BMD of the lumbar spine and total body density and body height SDS. Predictors were mean serum Phe, coefficient of variation of serum Phe values, maximum serum Phe, mean serum Tyr and mean serum Phe/Tyr ratio, sex and age at DXA measurement. For the evaluation of model quality, a coefficient *R*^2^ was used. The coefficient of serum Phe values variation (CV) was defined as a quotient of SDS (STDPhe) and mean serum Phe (MEANPhe): CV  =  STDPhe/MEANPhe.

**Table 3:** Metabolic predictors of clinical characteristics of the treated patients.

	Predictor	β	*P*-value
Body height SDS	Mean serum Phe	−0.562	<0.001
*R*^2^ = 0.264	CV serum Phe^a^	−0.262	0.009
*P* = <0.001			
BMI SDS	CV serum Phe^a^	−0.211	0.042
*R*^2^ = 0.073	Sex^b^	0.210	0.043
*P* = 0.014			
Proportion abdominal fat	Sex^b^	−0.260	0.032
*R*^2^ = 0.101	Age at DXA	0.251	0.039
*P* = 0.014			
Proportion whole-body fat	Sex^b^	−0.317	0.010
*R*^2^ = 0.086			
*P* = 0.010			
Proportion abdominal fat/Proportion whole-body fat ratio	Age at DXA	0.570	<0.001
*R*^2^ = 0.431	Mean serum Tyr	−0.226	0.025
*P* = <0.001			
B12	CV serum Phe^a^	0.305	0.003
*R*^2^ = 0.106	Sex^b^	0.204	0.045
*P* = 0.003			
Selenium	CV serum Phe^a^	−0.239	0.020
*R*^2^ = 0.108	Mean serum Tyr	−0.252	0.014
*P* = 0.003			
Zinc	Mean serum Phe/Tyr ratio	0.332	0.002
*R*^2^ = 0.093			
*P* = 0.002			
Mean BMD T- and Z-scores (L1–L4)	Age at DXA	−0.369	<0.001
*R*^2^ = 0.127			
*P* < 0.001			
Mean BMD T- and Z-scores (total body)	Age at DXA	−0.393	0.001
*R*^2^ = 0.141			
*P* = 0.001			

^a^Coefficient of serum Phe values variation (CV) was defined as a quotient of s.d. and mean serum Phe (x̅); ^b^Positive β indicates positive association with male sex and negative β indicates positive association with female sex.

β, standardized regression coefficient; B12, vitamin B12; BMD, bone mineral density; CV, coefficient of variation; DXA, densitometry; L1–L4, first to forth lumbar vertebra; Phe, phenylalanine; *R*^2^, proportion of variation in dependent variable explained by regression model; SDS, standard deviation score; Tyr, tyrosin.

As we were analysing a national cohort, the demographic characteristics of the groups could not be manipulated. To diminish the influence of possible age and sex differences, the following variables – BMD, BMI and height – were statistically compared using exclusively normalized data (T- and Z-scores for BMD and SDS values for BMI and height). In addition, to estimate the importance of sex and age of patients, we performed correlation analysis by computing Pearson correlation coefficients between age of patients at DXA measurements with all the predictors and tested the differences of the dependent variables according to sex by using independent samples *t*-tests.

The statistical analyses were performed using the SPSS software package (IBM SPSS Statistics, ver.23).

## Results

The comparison of demographic, anthropometric, metabolic, nutritional indicators and BMD between treated and untreated patients and between subgroups in treated patients (cPKU, non-cPKU) are stated in Tables 1 and 2, respectively. The patients’ age and age at DXA measurement were significantly higher in treated patients as compared to the untreated group. The patients with cPKU were on average older as compared to the non-cPKU group, but the difference was not significant. There was a male preponderance in the untreated group, but not in other groups.

Prior to regression analysis we wanted to analyze the importance of age and sex in the treated group of patients. Therefore, we performed the correlation analysis of age at DXA measurement with all the predictors of the regression analyses. The correlations between age at DXA measurement with mean Phe (*r* = 0.684, *P* < 0.001) and mean Phe/Tyr ratio (*r* = 0.586, *P* < 0.001) were high, while correlations with maximum Phe (*r* = 0.428, *P* < 0.001), coefficient of variation of serum Phe values (*r* = −0.355, *P* < 0.001) and mean Tyr (*r* = −0.280, *P* = 0.006) were estimated as medium.

To estimate the importance of sex, the statistical analysis of differences of BMI SDS, the proportion of abdominal fat, the proportion of whole-body fat, the proportion of abdominal fat/proportion of whole-body fat ratio, serum vitamin B12 and selenium, Z-scores and T-scores for mean BMD of the lumbar spine and body height SDS according to sex were additionally performed. Statistically significant differences according to sex were found in the BMI SDS (*P* = 0.031), the proportion of abdominal fat (*P* = 0.033), the proportion of whole-body fat (*P* = 0.008) and in serum vitamin B12 (*P* = 0.047). All other differences were not significant.

According to these results, we included the age and sex of patients in all the following regression analyses. The results of a multiple stepwise linear regression of clinical parameters in the whole treated group are listed in Table 3. The reported results were just variables, which were statistically significant in the regression analyses.

There was a significantly lower body height SDS in the treated group vs untreated group (*P* < 0.001) and group cPKU vs non-cPKU (*P* < 0.001). The significance disappeared when height SDS was compared in patients older than 18 years in treated vs untreated groups (*P* = 0.425). There was, however, a significant difference in final height in cPKU compared to non-cPKU treated adult patients (*P* = 0.012). Furthermore, the mean serum Phe and variability in serum Phe negatively predicted body height SDS (β = −0.562, *P* < 0.001; β = −0.262, *P* = 0.009) in the treated group. Nevertheless, the mean final height in the treated group was not different from the general population (Tables 1 and 2).

The proportion of the overweight in the treated group was 26.6% (5.3% obesity) and 10% (5% obesity) in the untreated group. BMI SDS and the proportion of abdominal fat were not statistically different among the groups; however, the variability in serum Phe negatively predicted BMI SDS (β = −0.211, *P* = 0.042). Interestingly, the proportion of whole-body fat was statistically higher in treated non-cPKU patients as compared to cPKU (*P* = 0.048). In multiple regression analysis of the treated group, abdominal and whole-body fat correlated with age and sex (Table 3), similarly as described in general populations (20, 21). Despite the fact that treated and untreated groups were unbalanced in sex and age, the differences between these two groups in body fat proportions were not statistically significant (Tables 1 and 2).

Despite the diet, mean serum Phe levels were significantly higher with increased disease severity, defined by a higher maximal Phe and lower Phe intake tolerance, in the treated group vs the untreated group and in cPKU vs non-cPKU treated patients. Nevertheless, mean serum Tyr levels were not significantly different among groups, reflecting adequate protein intake in the treated group (Tables 1 and 2).

Serum zinc and selenium levels were measured only in treated patients and were not significantly different between the cPKU and non-cPKU patients, but vitamin B12 levels were significantly higher in cPKU group comparing to the rest of the treated patients (Table 2). Nevertheless, mean serum Phe/Tyr ratio positively predicted serum zinc values (β = 0.332, *P* = 0.002) in treated patients. Variability of serum Phe values and mean serum Tyr negatively predicted serum selenium values (β = −0.239, *P* = 0.020; β = −0.252, *P* = 0.014) in treated patients (Table 3).

There was no statistically significant difference in lumbar spine BMD or total body BMD between the groups (Tables 1 and 2). The only significant predictor of BMD was age at measurement.

## Discussion

Our study is one of the largest published national populations of early diagnosed and treated patients with HPA with complete age and gender corrected data in pediatric and adult age, which was analyzed for the demographic, anthropometric, metabolic, nutritional indicators and BMD (9). This study is one of the rare studies that compared the treated HPA patients to untreated HPA cohort with bHPA; other studies compared growth and BMI outcomes (22) and vitamin and mineral status (23) in similar groups of patients.

The mean body height of the bHPA participants was higher in comparison to the treated HPA patients, but the significance disappeared when height SDS was compared in patients older than 18 years, suggesting that patients despite dietary treatment gained a normal final height, which was similar to healthy peers. Nevertheless, the significantly lower body height in patients with cPKU compared to non-cPKU patients was evident regardless of age. Furthermore, the mean serum Phe, indicating dietary compliance, and variability in serum Phe, both negatively predicted body height in the treated group. Our study supports the findings of other authors that, nowadays, early treated HPA patients can achieve normal height (22, 24), but the final height of cPKU patients may be compromised (25, 26). The improved dietary management with a more adequate composition of amino acid mixtures, availability of low protein foods and differences in diet patterns probably explains a better growth in non-cPKU group, but these treatment advantages may not be sufficient in patients with cPKU.

The link between PKU and the tendency to overweight is discussed in many studies (27, 28) and a trend toward increased body weight in patients with PKU in both sexes was described (28, 29, 30). Because of a restriction of natural proteins, the diet is rich in carbohydrates and fat, which could predispose PKU patients to obesity (31). Obesity could also be a result of poor adherence to the diet, decreased activity levels or an underlying condition (27). However, not all recent studies have confirmed this. In Portugal, UK, Austria and Spain, overweight and obesity prevalence in patients with PKU was similar to the control population (11, 22, 29). Similarly, in our study there was no significant difference in BMI between the groups. The variability of Phe, as an indicator of long term dietary compliance, was a negative predictor of BMI. Furthermore, as compared to the general population of Slovenian children, where overweight and obesity are expected in 15.7% and 5.2%, respectively (32), there was a higher prevalence of overweight, but not obesity in treated patients, but not in participants with bHFA.

No statistically important differences between the groups and no effects of parameters other than sex and age on whole-body and abdominal fat were identified in our cohort. The proportion of whole-body fat was even lower in patients with cPKU compared to non-cPKU. Additionally, the ratio between the abdominal and total fat was negatively predicted by mean serum Tyr, an indicator of amino acid supplementation. In a body composition study, Albersen and colleagues described a statistically significantly increased mean proportion of whole-body fat in PKU patients compared to the control group, especially in girls over 11 years of age (33). On the contrary, studies in Portugal and Austria are consistent with our findings (10, 11).

No difference was observed in fat composition or BMI SDS in PKU as compared to bHFA group, which could be a positive consequence of improved nutrition of PKU patients over the last decades. Of note, serum Phe variability covers less than 7.3% of the BMI SDS variability. The patients with cPKU had the highest risk of poor diet adherence; however, the proportion of whole-body fat was higher in non-cPKU with less limited diet but with a better selection of various snacks and pre-prepared food.

Most studies confirm that bone is affected in PKU patients, but the conclusions about the biological causes and risk factors are inconsistent (34, 35). Several explanations for reduced BMD in patients with PKU have been proposed. Severely restricted protein diet with long-standing deficiency in protein, calcium, phosphate, vitamin D or trace elements (8, 36), low adherence to diet with insufficient intake of Phe free protein supplement (37), inadequate control of plasma phenylalanine level with possible toxic effect of the high Phe levels and disease itself on bone turnover (34, 35, 38) and lower physical activity (39) were suggested. The recent systemic reviews and meta-analyses have failed to confirm the consistent relationship between these risk factors and BMD (9, 40).

In our study, untreated HPA patients had normal BMD and we speculate that bHPA itself did not negatively affect bone turnover. In the treated group, a stricter diet with a greater protein restriction had no negative impact on BMD. The only predictor of worse BMD outcome was older age. No effect of age or Phe level on BMD was seen in a study, which analyzed adult patients only (36); however, our cohort consisted of adults and minors and therefore comprised age differences in management. Another study analyzing children and adolescents divided by pubertal stage and Phe levels demonstrated negative effect of age and higher Phe levels on bone health. (38) Of note, in our cohort, age highly correlated with mean Phe values, likely due to longer periods of less strict diet and changes in contemporary dietary approaches in recent years. Consequently, compliant patients with adequate protein substitution may have higher BMD. As shown in another study, the variability of serum Phe negatively predicts serum selenium and selenium was suggested as a contributing factor for osteopenia in children with PKU (39). Plasma selenium represents an adequate biomarker for selenium status in patients on diets (41). Selenium deficiency may activate bone-resorbing (42). Normal selenium measured in our cohort possibly contributed to normal BMD in our treated patients. However, studies assessing medical food intake in PKU patients and BMD are inconsistent, and while some report no correlation with BMD (8, 36), a recent publication has shown compliance with medical food prescription to be a major predictor of BMD (12). While BMD, as a complex variable, depends on many factors, our predictors also described a smaller part of variability.

Bone status in the majority of the studies has been analyzed primarily by DXA, which measures only areal BMD and neglects the bone size comparing to quantitative computed tomography (CT) (35, 37). Patients with cPKU in our study had lower body height, but BMD was not significantly lower compared to the untreated group, regardless of the missing adjustment of BMD data for height. A significant difference in the mean age at DXA measurement in the treated and untreated group might be a limitation of our study, but the reported Z-scores and T-scores lowered the risk of bias. Our data were in line with the recent reports on normal mean BMD in early treated patients, but the comprehensive analysis of impaired muscle-bone unit by quantitative CT opens the questions about the primary and secondary bone disease in PKU (8, 35).

## Conclusions

Low protein dietary treatment in PKU patients delayed growth in childhood, but the patients gained a normal final height, which was similar to healthy peers. Well-controlled dietary treatment with adequate nutrition had no significant consequences on BMI, body composition and BMD. Untreated bHPA had no negative consequences on the observed parameters.

## Declaration of interest

The authors declare that there is no conflict of interest that could be perceived as prejudicing the impartiality of the research reported.

## Funding

The study was supported in part by the Slovenian National Research Agency grant P3-0343.
